# Immune-related gene-based prognostic index for predicting survival and immunotherapy outcomes in colorectal carcinoma

**DOI:** 10.3389/fimmu.2022.944286

**Published:** 2022-12-13

**Authors:** Zhongqing Liang, Ruolan Sun, Pengcheng Tu, Yan Liang, Li Liang, Fuyan Liu, Yong Bian, Gang Yin, Fan Zhao, Mingchen Jiang, Junfei Gu, Decai Tang

**Affiliations:** ^1^ School of Chinese Medicine, School of Integrated Chinese and Western Medicine, Nanjing University of Chinese Medicine, Nanjing, Jiangsu, China; ^2^ Affiliated Hospital of Nanjing University of Chinese Medicine, Nanjing, Jiangsu, China; ^3^ Laboratory of New Techniques of Restoration & Reconstruction of Orthopedics and Traumatology, Nanjing University of Chinese Medicine, Nanjing, Jiangsu, China; ^4^ Laboratory Animal Center, Nanjing University of Chinese Medicine, Nanjing, Jiangsu, China

**Keywords:** colorectal carcinoma, immune-related gene prognostic index (IRGPI), tumor immune microenvironment (TIM), immune-related gene (IRG), immune checkpoint inhibitor (ICI)

## Abstract

**Introduction:**

Colorectal cancer shows high incidence and mortality rates. Immune checkpoint blockade can be used to treat colorectal carcinoma (CRC); however, it shows limited effectiveness in most patients.

**Methods:**

To identify patients who may benefit from immunotherapy using immune checkpoint inhibitors, we constructed an immune-related gene prognostic index (IRGPI) for predicting the efficacy of immunotherapy in patients with CRC. Transcriptome datasets and clinical information of patients with CRC were used to identify differential immune-related genes between tumor and para-carcinoma tissue. Using weighted correlation network analysis and Cox regression analysis, the IRGPI was constructed, and Kaplan–Meier analysis was used to evaluate its predictive ability. We also analyzed the molecular and immune characteristics between IRGPI high-and low-risk subgroups, performed sensitivity analysis of ICI treatment, and constructed overall survival-related receiver operating characteristic curves to validate the IRGPI. Finally, IRGPI genes and tumor immune cell infiltration in CRC model mice with orthotopic metastases were analyzed to verify the results.

**Results:**

The IRGPI was constructed based on the following 11 hub genes: ADIPOQ, CD36, CCL24, INHBE, UCN, IL1RL2, TRIM58, RBCK1, MC1R, PPARGC1A, and LGALS2. Patients with CRC in the high-risk subgroup showed longer overall survival than those in the low-risk subgroup, which was confirmed by GEO database. Clinicopathological features associated with cancer progression significantly differed between the high- and low-risk subgroups. Furthermore, Kaplan–Meier analysis of immune infiltration showed that the increased infiltration of naïve B cells, macrophages M1, and regulatory T cells and reduced infiltration of resting dendritic cells and mast cells led to a worse overall survival in patients with CRC. The ORC curves revealed that IRGPI predicted patient survival more sensitive than the published tumor immune dysfunction and rejection and tumor inflammatory signature

**Discussion:**

Thus, the low-risk subgroup is more likely to benefit from ICIs than the high-risk subgroup. CRC model mice showed higher proportions of Tregs, M1 macrophages, M2 macrophages and lower proportions of B cells, memory B cell immune cell infiltration, which is consistent with the IRGPI results. The IRGPI can predict the prognosis of patients with CRC, reflect the CRC immune microenvironment, and distinguish patients who are likely to benefit from ICI therapy.

## Introduction

According to the Global Statistical Report on Cancer in 2020 (1), colorectal carcinoma (CRC) is one of the most common malignant tumors, ranking third in morbidity and second in mortality. More than 1.9 million new CRC cases and 935,000 CRC-related deaths were estimated to occur in 2020. The 5-year survival rate of patients with metastatic CRC is low at approximately 14% (2), and approximately 50% of patients who receive treatment develop metastases (3, 4).

Immunotherapy is a cutting-edge option for treating cancer that involves stimulation of specific immune responses to utilize the body’s own immune system to suppress and kill tumor cells, thereby reducing tumor recurrence and metastasis. Immune checkpoint inhibitors (ICIs) are promising agents for treating a variety of solid tumor malignancies such as melanoma and lung cancer. Pembrolizumab and nivolumab are ICIs targeting programmed cell death protein 1 and have both been approved by the U.S. Food and Drug Administration for the treatment of microsatellite instability-high/DNA mismatch repair-deficient CRC (5, 6). However, this tumor type accounts for only 5% of metastatic CRCs, and the remaining patients show poor responses to ICI (7, 8). Various factors, including immunoassay sites and the tumor immune microenvironment (TIME), affect the effectiveness of ICIs, and the analysis of TIME can lead to the development of methods for improving the reactivity to immunotherapy (9).

**Figure 1:**
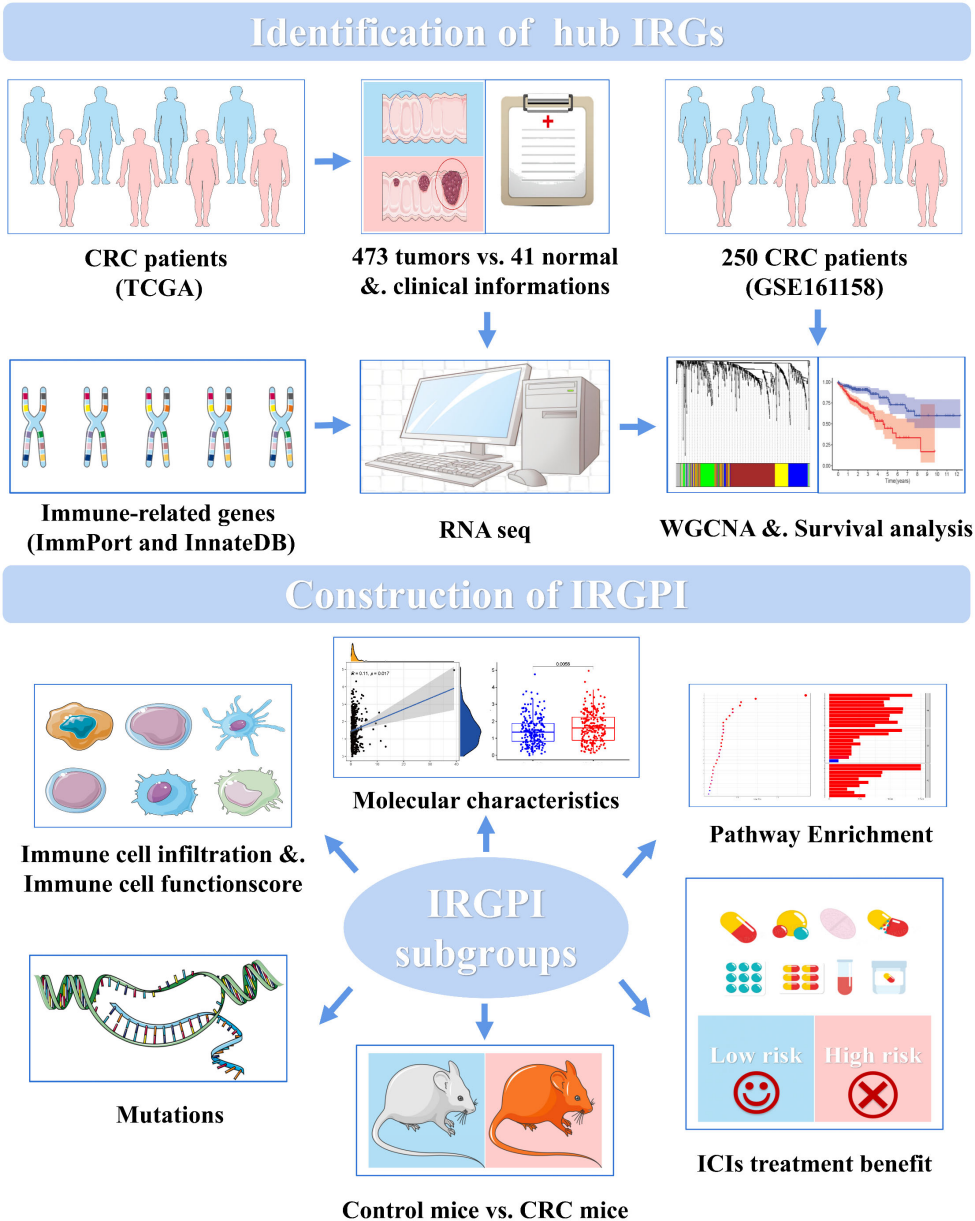
CRC IRGPI experimental technical roadmap.

Only few biomarkers have been identified for predicting patient prognosis. Therefore, more biomarkers that can reflect the benefit of immunotherapy as a clinical reference for predicting the survival and prognosis of patients with CRC are needed. In this study, we constructed a CRC-related prognostic marker using 11 genes that could predict the prognosis of immunotherapy, as shown in **Figure 1**. Based on the differential immune-related genes in the transcriptome data of patients with CRC in TCGA (The Cancer Genome Atlas), we identified immune-related hub genes and weighting coefficients related to patient prognosis, constructed the immune-related gene prognostic index (IRGPI), and verified its power reliability using multiple datasets. We then characterized the molecular and immune signatures between high- and low-risk subgroups determined using the IRGPI, examined their prognostic power in patients following immunotherapy, and compared them with other biomarkers, tumor immune dysfunction and rejection (TIDE), and tumor inflammatory signature (TIS). To simulate the characteristic environment in patients with CRC, we established an animal model of CRC to verify the prediction ability of the IRGPI. Our results suggest that IRGPI is a promising prognostic biomarker in patients being administered conventional immunotherapy and immunotherapy.

## Materials and methods

### Collection of sample information

As the test group, transcript data and clinicopathological information were downloaded from the TCGA database (https://portal.Gdc.cancer.gov/), including 41 cases of para-tumors, 473 cases of CRC tumors, and 452 clinical cases. The TCGA data matrix was used to establish IRGPI in the following series of studies.

As the train group, survival and transcriptional data for 250 CRC cases were downloaded from the GEO database (https://www.ncbi.nlm.nih.gov/geo/). The transcript dataset GSE161158 was uploaded in November 2020 by the Moffitt Cancer Research Center, University of Miami (10). The GEO survival analysis was used to validate the results of IRGPI.

Lists of immune-related genes were downloaded from ImmPort (https://www.immport.org/home) and Innate DB (https://www.innatedb.ca/). KEGG (http://www.gsea-msigdb.org/gsea/index.jsp) gene sets and all Gene Ontology (GO) gene sets were used as gene symbols. Gene mutation information was downloaded from the cBioPortal (http://www.cbioportal.org/).

### Identification of immune-related differential genes

Limma package was used to analyze the differential expression of immune-related genes in para-tumor and tumor tissues. The ratio of expression between two samples (groups) [Log2fold-change (FC) = log2(treat mean/control mean)] and the *p*-value were calculated. A Log2FC >0 indicated that the gene is upregulated in the tumor tissue, whereas a Log2FC<0 indicated that the gene is downregulated in the tumor tissue. The false discovery rate was obtained by correcting the *p*-value (|Log2FC| >1 and false discovery rate<0.05). Differential immune-related genes (IRGs) identified using TCGA were analyzed for GO terms and KEGG pathway enrichment. The filter was adjusted to *p*< 0.05, and circles, bar plots, and bubble plots were drawn.

### Weighted gene co-expression network analysis to identify immune-related hub genes

The differential IRG dataset was selected to eliminate samples with data fluctuation and free and missing data, and the mean value of repeated data was determined. The Pearson correlation coefficient between any two genes was calculated; if the coefficient was higher than the threshold of 0.3, the two genes were considered as similar. The weighted value of the correlation coefficient was used in the analysis, taking the IRG correlation coefficient to the 20th power so that the connection between the genes obeyed the scale-free network distribution (11). These data were then converted into a topological matrix that described the degree of association between genes using a topological overlap metric. The genes were clustered using 1 − topological overlap metric as the distance, and a dynamic pruning tree was constructed to identify the modules (12). Finally, five modules were identified by setting the merge clipping threshold to 0.25. Univariate Cox regression analysis was performed on module (blue, brown, and yellow) genes significantly associated with CRC, and the correlation between the module genes and overall survival (OS) was determined. Thirty-four immune-related hub genes showing significant associations with survival were selected for further analysis.

### Establishment and verification of IRGPI

For the 34 immune-related hub genes, multivariate Cox regression analysis was used to construct the IRGPI. The risk score of each patient was obtained by the coefficient of multiplying the expression data of IRGs, and the patients were divided into high- and low-risk groups according to the median value of the risk score. The prognosis of subgroup patients defined using the IRGPI was assessed using the Kaplan–Meier (K–M) survival curve and log-rank test of TCGA and GEO cohorts. Prognostic univariate and multivariate Cox regression analyses were performed for age, sex, stage, and risk score to determine whether the risk score was affected by other factors. To detect associated genetic alterations, somatic mutations in patients between the high- and low-risk subgroups were analyzed using the maftools package in R software (The R Project for Statistical Computing, Vienna, Austria).

### Assessment of immune cell infiltration and immune characteristics

CIBERSORT was used to calculate the immune cell infiltration of samples from the TCGA dataset (13). According to the risk score, differences in immune cell infiltration between the high- and low-risk subgroups were counted, a boxplot was obtained, and the survival curves of the two groups were plotted. The immune function scores of samples in the high- and low-risk subgroups were calculated to obtain immune-related function scores for each sample. Higher scores indicated weaker immune function and *vice versa*. In addition, IC gene expression and risk analyses were performed in the high- and low-risk groups using the IRGPI.

### IRGPI and TIDE score

The TIDE score table, including the TIDE score, exclusion immune rejection, and dysfunction, was obtained from the TIDE database (http://tide.dfci.harvard.edu/) according to TCGA transcriptome files. Wilcoxon test was performed on the TIDE scores in the high- and low-risk subgroups, and a violin plot was drawn according to the results. The OS-related receiver operating characteristic (ROC) curves of TIDE, TIS, and IRGPI were plotted to compare the predictive powers of these values. The ROC curve of IRGPI related to OS at 1, 2, and 3 years was drawn; a larger area under the curve indicated that the model had higher prediction sensitivity.

### IRGPI gene expression in the CRC murine model

Specific pathogen-free BALB/c male mice, 6–8 weeks old with a body mass of 20 ± 5 g, were purchased from the Huaxing Experimental Animal Farm of Huiji District (Zhengzhou City, China), under the experimental animal license No. SCXK (Yu) 2019-0002. All animal experiments were approved by the Experimental Mouse Ethics Committee of the Nanjing University of Traditional Chinese Medicine (No. 202010A026).

The mice were randomly divided into the control group and the CRC model group, with 10 mice per group. Five BALB/c male mice were used as tumor-bearing mice, into which 1 × 10^7^ CT26 cells were subcutaneously injected into the left axilla and sacrificed 1 week later. The subcutaneous tumor was removed under sterile conditions, placed in sterile phosphate-buffered saline, and divided into several 1-mm^3^ masses. Under sterile conditions, the two groups of mice were dissected to expose the colon; the 1-mm^3^ tumor mass was fixed to the colon of the CRC model group with tissue glue, whereas the control group was not fixed, and then the abdomen from mice in the two groups was sutured. After 3 days of postoperative recovery, the mice were weighed, and micro-computed tomography scanning was performed on day 26 (under isoflurane respiratory anesthesia). On day 27, the mice were sacrificed under anesthesia with 2% sodium pentobarbital. Total RNA was extracted from the colon of the control group and tumor tissue of the CRC model group using a FastPure Cell/Tissue Total RNA Isolation Kit (Vazyme, Nanjing, China, Cat#RC101-01). The RNA was reverse-transcribed into cDNA using HiScript^®^ III RT SuperMix for quantitative PCR (Vazyme, Cat#R323-01), and then real-time PCR was performed to detect the expression of IRCPI genes in each group using Blastaq™ Green 2× qPCR MasterMix (abm, Richmond, British Columbia, Canada, Cat#G891). The primer sequences are listed in **Table S4**.

### Immune infiltration in the CRC murine model

The liver, colon, tumor, and mesentery of paraffin-embedded mice were sectioned, stained with hematoxylin–eosin, and photographed using an upright white light photographic microscope (Eclipse Ci-L, Nikon, Tokyo, Japan).

TIME immune cells were detected using flow cytometry. Peripheral blood mononuclear cells were extracted using RBC lysate (Cat#FMS-RBC500, FcMACS, Nanjing, China). At least 5 × 10^6^ cell suspensions (100 μl) were incubated with FC blocker at 4°C for 10 min and then anti-human/mouse CD11b FITC antibody (Cat#03221-50, PeproTech, Rocky Hill, NJ, USA), PE-Cy™7 rat anti-mouse CD86 antibody (Cat#560582, BD Pharmingen™, San Diego, CA, USA), and Alexa Fluor^®^ 488 anti-mouse CD206 antibody (Cat#141710, BioLegend, San Diego, CA, USA) were used to detect macrophages. Alexa Fluor^®^ 488 anti-mouse CD19 antibody (REF#11-0193-81, Invitrogen, Carlsbad, CA, USA) and PE/Cy7 anti-mouse/rat/human CD27 antibody (Cat#124216, BioLegend) were used to detect B cells. Anti-mouse CD4 APC-cyanine7 (Cat#06122-87, PeproTech), anti-mouse CD8a FITC antibody (Cat#10122-50, PeproTech), anti-mouse CD25 APC antibody (Cat#07312-80, PeproTech), and anti-mouse/rat FOXP3 PE antibody (Cat#83422-60, PeproTech) were used to detect T cells. The cells were detected on an Amnis FlowSight flow cytometer (Merck Millipore, Billerica, MA, USA), and immunocyte subsets were analyzed using the IDEAS software (Merck Millipore). **Figure S7** shows the strategy used to analyze the IRGPI immunocyte subsets using flow cytometry.

### Statistical methods

Independent *t*-tests were performed to compare continuous variables between the two groups. Data for various clinicopathological factors were analyzed using chi-square test. The TIDE scores between the groups were compared using Wilcoxon test. Univariate survival analysis was performed using K–M survival analysis and log-rank test. Univariate and multivariate Cox regression analyses were performed using the R package “survival” with hazard ratios and 95% confidence intervals. *p*< 0.05 indicated a significant difference between the two groups. The software Strawberry-perl-5.30.2.1, Rx64 4.1.0 (R Project), and GraphPad Prism 8 (GraphPad, Inc., La Jolla, CA, USA) were used in these analyses.

## Results

### CRC differential immune-related genes

A total of 7,780 differentially expressed genes (473 tumors versus 41 para-tumor samples) were identified in TCGA-differential expression analysis, among which 5,502 and 2,278 genes were up- and downregulated, respectively, in tumor samples compared to the genes in para-tumor samples (**Tables S1** and **S2**). By intercrossing these genes with immune-related genes obtained from ImmPort and InnateDB, 649 differential immune-related genes were obtained, among which 256 and 393 genes were up- and downregulated, respectively, in tumor samples compared to those in para-tumor samples (**Figure S1A**).

Enrichment analysis of 649 immune-related differentially expressed genes screened from the TCGA dataset revealed significant correlations in 1,295 GO terms (*p*< 0.001) and 66 KEGG pathways (*p*< 0.05). The top 30 GO terms and KEGG pathways are shown in **Figure S1B**. The top three pathways enriched in GO analysis were humoral immune response, complement activation, and classical pathway (**Figure S2A**). The top three pathways enriched in KEGG analysis were cytokine–cytokine receptor interaction, viral protein interaction with cytokine and cytokine receptor, and chemokine signaling pathway (**Figure S2B**).

### Construction of IRCPI with 11 CRC immune-related hub genes

**Figure 2:**
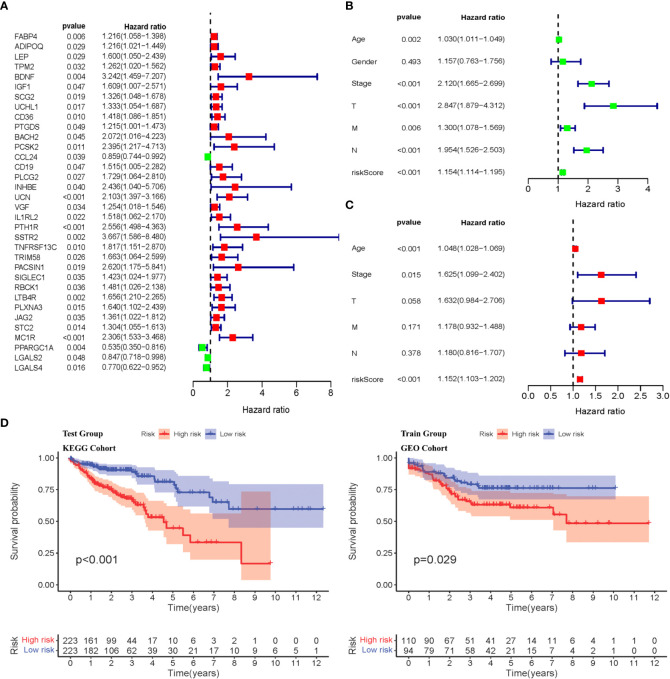
Identification of immune-related diffrentional genes *via* Cox regression and K-M analysis of IRGPI subgroups. **(A)** Univariate Cox analysis of 34 immune-related hub genes (*p*< 0.05). **(B, C)** Univariate and multivariate Cox regression analysis on IRGPI riskscore and other clinicopathologic variables. **(D)** Kaplan–Meier survival analysis between IRGPI subgroups in the TCGA cohort and GEO cohort (*p*< 0.05).

Weighted gene co-expression network analysis was used to analyze immune-related differential genes (*n* = 649), and immune-related hub genes were identified (**Figure S3A**). The logarithm log(*k*) of the node with connectivity *k* and logarithm log of the node probability [*P*(*k*)] were negatively correlated, with a correlation coefficient >0.9 and an optimal soft threshold of 4. A total of 649 genes were assigned to the five modules. According to the Pearson correlation coefficients between modules and sample features, the blue, brown, and yellow modules were closely associated with CRC and positively correlated (**Figure S3C**). Genes in these modules were selected for further analyses. Thirty-four hub genes were significantly associated with OS according to univariate Cox regression analysis (*p*< 0.05) (**Figure 2A**, **Table S3**). Next, multivariate Cox regression analysis was performed, and 11 hub genes were obtained to establish prognostic indicators. This result was validated in the K–M analysis, as shown in **Figure S3D** (*p*< 0.05). Specifically, the IRGPI risk score was calculated using the gene expression levels multiplied by the weights of the 11 genes, as shown in **Table 1**.

**Table 1:** The 11 immune-related hub genes used to compute the IRGPI risk score.

ID	Coef	HR	HR.95L	HR.95H	*p*-value
**ADIPOQ**	−0.2869	1.21605	1.02064	1.44886	0.02863
**CD36**	0.77205	1.41784	1.08580	1.85140	0.01033
**CCL24**	−0.15359	0.85923	0.74388	0.99247	0.03914
**INHBE**	1.041035	2.43576	1.03974	5.70616	0.04039
**UCN**	0.584717	2.10328	1.39728	3.16602	0.00037
**IL1RL2**	0.310954	1.51794	1.06197	2.16971	0.02203
**TRIM58**	0.599037	1.66316	1.06424	2.59914	0.02553
**RBCK1**	0.322746	1.48107	1.02588	2.13822	0.03605
**MC1R**	0.526967	2.30592	1.53315	3.46820	0.00006
**PPARGC1A**	−0.78226	0.53452	0.35002	0.81627	0.00373
**LGALS2**	−0.2145	0.84689	0.71843	0.99832	0.04771

### Molecular characteristics of CRC-related high- and low-risk IRGPI subgroups

The IRGPI was established by multiplying the expression data for the hub IRGs by the multivariate Cox regression coefficients, as follows: $risk\;score=\sum_{n=11}^{1}\left(gene\;expression\;data\times coef\right)$ . Based on the median risk score, the samples were divided into high- and low-risk groups. There was a significant difference in the survival period between high- and low-risk patients (*p*< 0.05), as shown in **Figure 2D**. Univariate and multivariate Cox regression analyses were performed on the risk scores and clinical traits of the two groups, respectively. The risk scores showed significant differences (*p*< 0.001) related to the stage, features/extent of the primary tumor (T), regional lymph node involvement (N), and distant metastases (M) (*p*< 0.001), but not sex or age (**Figures 2B, C**). We then explored the signatures of 34 immune-related hub genes. As shown in **Figure S4A**, in 28.32% of the 399 samples, 34 immune-related hub genes showed amplifications, deep deletions, and missense mutations. As we all know, targeted somatic mutation (TSM) reflects the immunotherapy resistance, and a higher TSM represents a worse immunotherapy outcome.

We selected the top 20 genes with mutation in the high- and low-risk groups (**Figure S4A**), and we found that the low-risk group had a higher altered rate. The mutation of *APC*, *TP53*, *TTN*, and *LRP2* was more common in the IRGPI-high subgroup, whereas the mutation of *SYNE1*, *PIK3CA*, *MUC16*, *FAT4*, *ZFHX4*, *RYR2*, *OBSCN*, *DNAH5*, *PCLO*, *LRP1B*, and *DNAH11* was more common in the IRGPI-low subgroup (**Figure S4B**). The result demonstrated the low-risk group with more universality of germline and somatic mutations in DNA mismatch repair (MMR) genes that have a chance to overcome the genomic instability.

KEGG pathway enrichment analysis of 11 IRGPI hub genes showed that the high-risk group was significantly enriched in cell adhesion, extracellular matrix, focal adhesion, and PPAR signaling pathways, which were associated with CRC progression and metastasis and indicated a worse prognosis (**Figure S4C**).

### Prediction of immune cell infiltration in the CRC microenvironment with IRGPI

**Figure 3:**
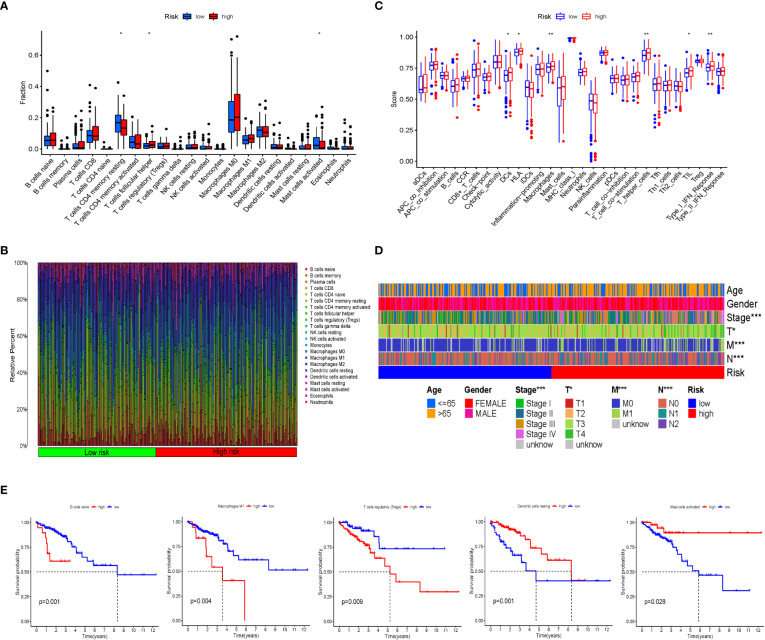
Immune cell infiltration and immune function scores between IRGPI subgroups. **(A)** Comparison of tumor immune cell infiltration between two IRGPI subgroups. Significant statistical differences between the two subgroups were assessed using the Wilcoxon test (*p< 0.05, **p< 0.01). **(B)** The proportions of TIME immune cells in different IRGPI subgroups. **(C)** Comparison of immune function score between two IRGPI-related CRC subgroups. Significant statistical differences between the two subgroups were assessed using the Wilcoxon test (*p< 0.05, **p< 0.01). **(D)** Clinicopathological information of the IRGPI-related CRC subgroups in the TCGA cohort. Age, gender, tumor stage, and T, M, and N are shown as patient annotations (*p< 0.05, **p< 0.01, ***p< 0.001). **(E)** Kaplan–Meier survival analysis of the TME cells and immune function IRGPI subgroups in the TCGA cohort.

CIBERSORT was used to analyze the infiltration of immune cells in the IRGPI subgroups. We detected more follicular helper T cells in the IRCPI high-risk subgroup, whereas CD4+ memory resting T cells and activated mast cells were more abundant in the low-risk subgroup (*p*< 0.05) (**Figure 3A**). Features associated with the immune landscape, including the clinicopathological features of different IRGPI subgroups, are shown in **Figures 3B, D**. Dendritic cells, human leukocyte antigen, macrophages, T helper cells, tumor-infiltrating lymphocytes (TIL), and type I interferon responses showed higher immune function scores (*p*< 0.05) in the high-risk IRCPI subgroup (**Figure 3C**). Immune function scores are correlated with the prognosis of patients with multiple tumors, and patients with high immune scores have a poorer prognosis compared to patients with low scores.

To further investigate whether the prognostic value of IRGPI is based on immune cell infiltration, we performed differential immune cell-related and immune function-related K–M analyses. As shown in **Figure 3E**, five types of immune cells were significantly associated with OS. Patients with more naïve B cells, macrophages M1, and T-cell regulatory (Treg) infiltration had poor OS, whereas patients with more resting dendritic cells and activated mast immune cell infiltration had a longer OS (*p*< 0.05).

### IRGPI is significantly associated with CRC progression

**Figure 4:**
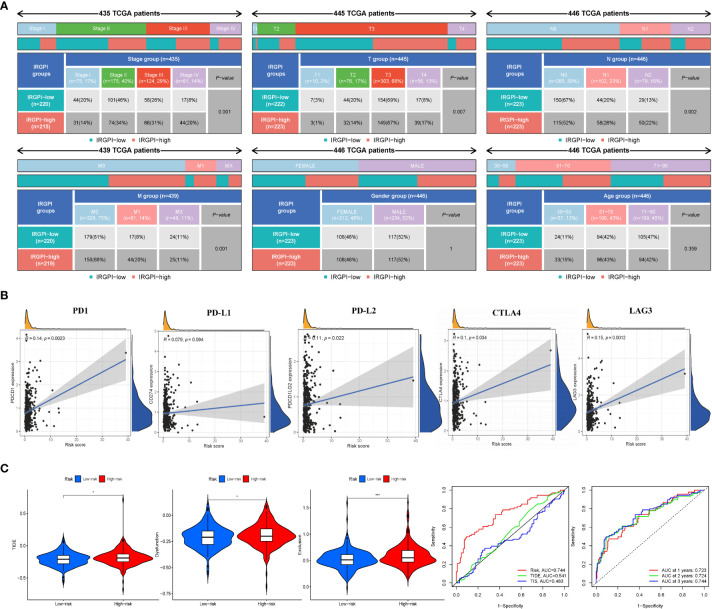
IRGPI signifcantly correlates with clinicopathological factors in CRC patients and the analysis of immunotherapy responses. **(A)** Heatmap and table showing the distribution of multiple clinicopathological factors in CRC patients between two IRGPI subgroups. **(B)** Scatter plots coordinated by IRGPI risk score with immune checkpoint PD-1, PD-L1, PD-L2, CTLA4, and LAG3, respectively. **(C)** TIDE, dysfunction, and T-cell exclusion score in different IRGPI subgroups. The scores between the two IRGPI subgroups were compared through the Wilcoxon test (*p< 0.05, ***p< 0.001). **(D)** Performance comparison between IRGPI risk, TIDE, and TIS in predicting 1-year OS in the TCGA cohort. Time-dependent ROC curve and AUC values in the TCGA cohort.

To explore the relationship between IRGPI and various clinicopathological factors, 445 patients in the high- and low-risk IRGPI subgroups were evaluated using chi-square test to determine the distribution of different clinical features, as shown in **Figure 4A**. Between the two subgroups, patients in the T, N, and M stages showed more severe cancer progression (*p*< 0.01), whereas age and sex were unrelated to progression. These results indicate that the prognostic value of IRGPI is related to CRC progression.

### IRGPI risk scores correlate with immunotherapy biomarkers

Some biomarkers are used in clinical immunotherapy, including programmed cell death protein-1 (PD-1), programmed death protein ligand 1/2 (PD-L1/2), cytotoxic T-lymphocyte-associated protein-4 (CTLA-4), and lymphocyte-activation gene 3 (LAG-3) (14). In addition to PD-L1/2 and CTLA4, CD27 and FOXP3 are biomarkers of activated B cells and Tregs, which can reflect the activity of immunocytes to some extent. We next explored the relationship between the IRGPI score and these biomarkers. As shown in **Figure 4B** and **Figure S6B**, the IRGPI score was positively correlated with PD-1, PD-L1/2, CTLA-4, and LAG-3. Pearson’s correlation coefficient (*R* value) between the IRGPI risk score and PD1 was 0.14, with a *p*-value of 2.3e-03 (PD-L1: *R* = 0.079, *p* = 9.4e-02; PD-L2: *R* = 0.11; *p* = 2.2e-02; CTLA-4: *R* = 0.1, *p* = 3.4e-02; LAG3: *R* = 0.15, *p* = 1.2e-03; CD27: *R* = 0.11, *p* = 1.7e-02; FOXP3: *R* = 0.12, *p* = 9.6e-03). In addition, the expression of IC genes in the high-risk subgroup was higher than that in the low-risk subgroup (*p*< 0.05), as shown in **Table S6**. Combined with the previous results of mutation, these results suggest that there were more immunosuppressive signals and tumor progression- and tumor metastasis-related signals in the high-risk group; meanwhile, there was more active immunity and damage repair in the low-risk group, which was consistent with the results of immune cell infiltration. In conclusion, the IRGPI low-risk group is more likely to develop an efficiently immune response from immunotherapy.

### IRGPI predicts benefits from immunotherapy

We used TIDE to assess the potential clinical efficacy of immunotherapy in different IRGPI subgroups. The TIDE score can evaluate the efficacy of immunotherapy for tumors, with a higher score representing a higher risk of immune evasion, suggesting that patients are less likely to benefit from ICI therapy (15). Our results show that the IRGPI high-risk subgroup had a higher TIDE score than the IRGPI low-risk subgroup (*p*< 0.05), with a higher T-cell dysfunction and exclusion score (**Figure 4C**). These results indicate that at-risk patients with a high IRGPI benefit less from ICI therapy compared to patients with a low IRGPI.

Analysis of the predictive power of IRGPI showed that its sensitivity was significantly higher than those of the TIDE and TIS scores (**Figure 4D**, area under the ROC curve: IRGPI = 0.744 > TIDE = 0.541 > TIS = 0.483). These results suggest that the IRGPI score is a suitable biomarker for predicting the immunotherapy response. In addition, the reliability of the IRGPI was determined using the time-dependent ROC curve (**Figure 4D**); we also tested the ROC curve using the TCGA dataset, as shown in **Figure 4D**. The areas under the 1-, 2-, and 3-year ROC curves were 0.723, 0.724, and 0.744, respectively, indicating that the IRGPI is useful for monitoring the survival rate.

### The CRC murine model verified the IRGPI predictive power

**Figure 5:**
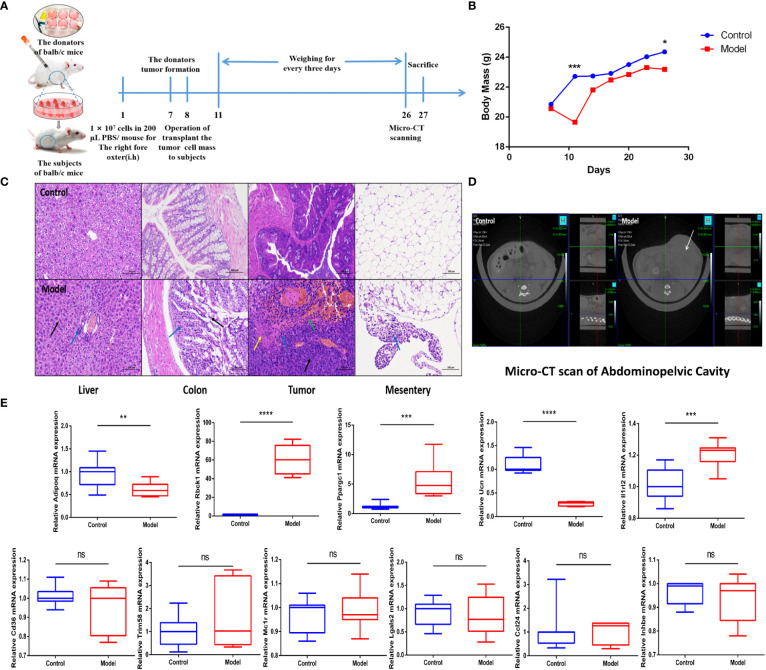
IRGPI gene expression situation in the identified murine CRC model. **(A)** Experimental scheme to establish the murine conlon transplant CRC metastasis model. **(B)** Curve of murine body mass change trends (*n* = 10, *p< 0.05, ***p< 0.001). **(C)** Inflammatory changes within liver, colon, tumor, and mesentery were measured by H&E staining (scale bar = 100 μm; the blue arrow points to the inflammatory cell infiltration; the black arrow points to the cellular damage; the yellow arrow points to the tumor cells nuclear abnormalities and cytoplasm eosinophilic; the green arrow points to the necrosis and hemorrhage). **(D)** Micro-CT scan images of abdominal cavity between control and model groups (the white arrow points to the tumor in abdominal cavity). **(E)** Relative IRGPI gene expression detection by RT-PCR between control and model groups. (Values are presented as 2^ΔΔCT^ mean ± SEM, *n* = 3 replicates per group, ns: not significant, *p< 0.05, **p< 0.01, ***p< 0.001, ****p< 0.0001.

To support the predictive power of the IRGPI, we established a CRC mouse model and tested IRGPI gene and immune cell infiltration in the TME. The weight changes in the two groups were recorded. Mice in the CRC model group showed weight loss at later stages of CRC (*p*< 0.05) (**Figure 5B**). We scanned the abdominal cavity using micro-computed tomography to determine the morphology of the CRC tumors (**Figure 5D**). The results confirmed sufficient maturity of the CRC murine model. Pathological sections of the liver, colon, tumor with colon cancer, and mesentery were obtained (**Figure 5C**). Compared to the normal group, mesentery lymph nodes in CRC model mice were degenerated and reduced, with focal infiltration of local lymphocytes. Compared with the colon tissue arranged in a compact and orderly manner, tumor cells in the colon from the model group showed nuclei atypia, a high nuclear–cytoplasmic ratio, inconspicuous nucleoli, more mitotic phase (black arrow), a large area of tissue necrosis (green arrow), deep staining and fragmentation of nucleus shrinkage, and enhanced eosinophilic cytoplasm (yellow arrow). The liver is the first metastatic organ affected by CRC. As shown in **Figure 5C**, compared with normal mice, the liver of CRC mice contained a large number of hepatocytes with granular degeneration and loose cytoplasm around the central vein, bile duct area, and liver parenchyma, with loose and light-stained granular cytoplasm (black arrow), along with a lymphocytic infiltrate around the local bile duct (blue arrow). IRGPI gene expression was detected in both groups. We observed higher expression levels of Il1rl2, Rbck1, and Ppargc1a in tumor tissues of the CRC group and higher expression levels of Adipoq and Ucn in the colon of the control group (**Figure 4E**). The risk coefficient of IRGPI is the comprehensive score calculated by the expression levels of 11 genes and their risk coefficients, through a complex process. It is worth noting that the IRGPI in the two kinds of mice may still have significance due to the distinction in the expression levels of the differential genes, in spite of the fact that there were no difference in some genes.

### Immune cell infiltration in the immune microenvironment of the CRC murine model has a negative impact on prognosis

**Figure 6:**
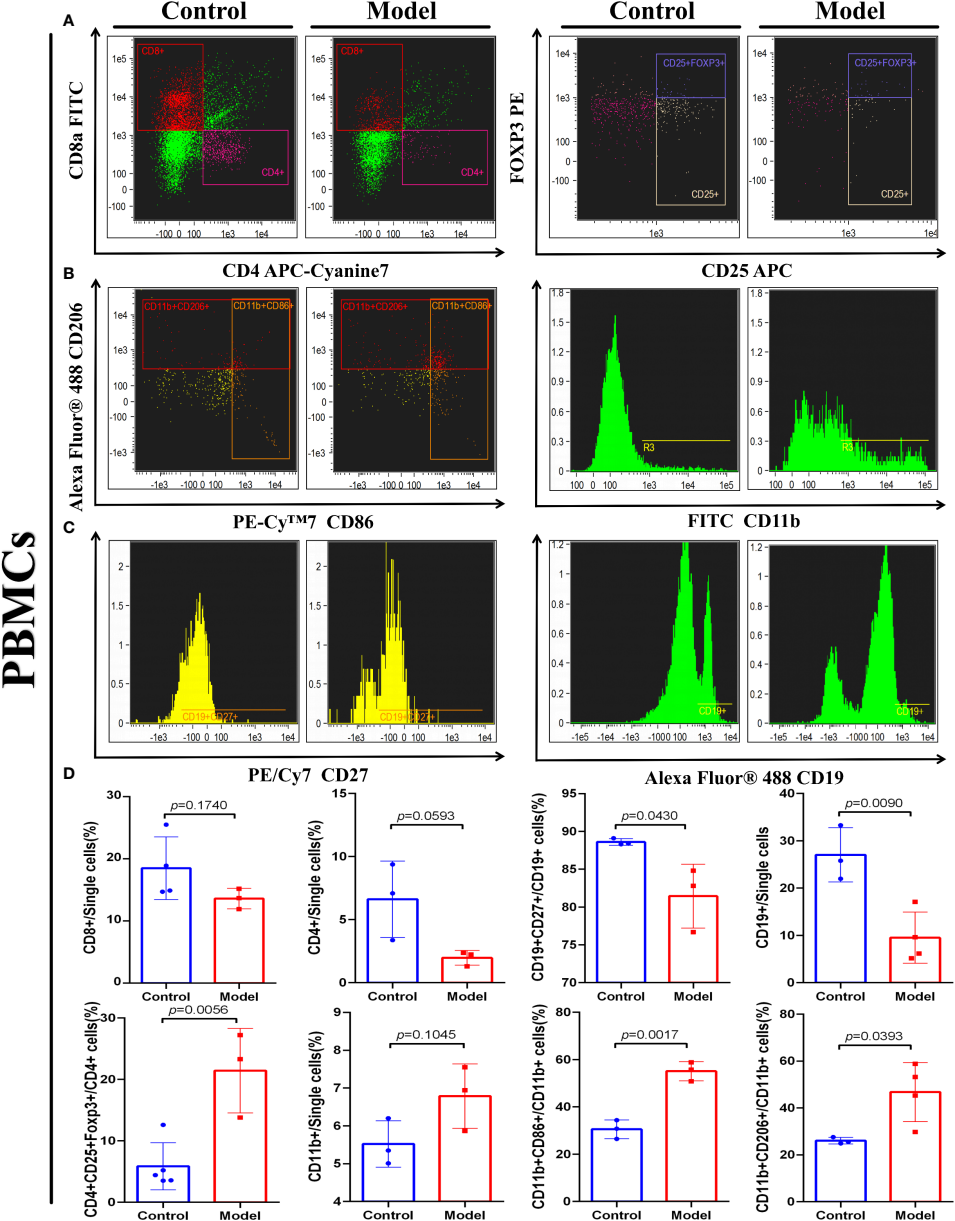
IRGPI-related immune cell discrepancy between CRC model mice with control mice. Flow cytometry determined the proportion of a series of immune-related cells that existed in murine peripheral blood. **(A)** T cells, **(B)** Macrophages, and **(C)** B cells. **(D)** Unpaired *t*-test analysis of the proportion of the immune-related cells: Cytotoxic T lymphocytes (CD8+), Helper T lymphocytes (CD4+) and Tregs cells (CD4+CD25+FOXP3+), B cells (CD27+), memory B cells (CD27+CD19+), Macrophages (CD11b+), M1 Macrophages (CD11b+CD86+), and M2 Macrophages (CD11b+CD206+) between control and the CRC model group. (Values are presented as mean ± SEM, *n* = 3 per group.).

To investigate whether the IRGP1 could predict changes in immune cell infiltration in CRC, we detected some immune cells in the peripheral blood of both groups of mice, as shown in **Figure 6**. An independent *t*-test was used to analyze the proportion of immune cells in the two groups. The proportions of Tregs CD4+CD8+FOXP3+ (*p* = 0.0056), M1 macrophages CD11b+CD86+ (*p* = 0.0017), and M2 macrophages CD11b+CD206+ (*p* = 0.0393) in CRC model mice were higher than those in the control group. The proportions of B cells CD19+ (*p* = 0.0090) and memory B cells CD19CD27+ (*p* = 0.0430) in the CRC group were significantly lower, whereas helper T lymphocyte CD4+ and cytotoxic T lymphocyte CD8+ cells in the CRC group tended to be lower than those in the control group.

These results indicate that changes related to T cells, B cells, and macrophages occur in the TIME of CRC, which is similar to the prediction results of the IRGPI. These results confirm the accurate prediction ability of the IRGPI.

## Discussion

In recent years, immunotherapy has been widely examined as a treatment for CRC, including ICIs, immunization, and adoptive T-cell therapy (16). An increasing number of ICI monotherapy or combination strategies is being designed to treat CRC. ICI therapy is an effective treatment for relapsed or refractory CRC (17). For example, PD-1 combined with a CTLA-4 blocker is clinically effective and well-tolerated in patients with advanced CRC having a defective DNA mismatch repair (18). However, CRC, like breast and ovarian cancers, is generally considered as a hypo-immunoreactive cancer (19), with limited infiltration of immune cells or extensive infiltration of immunosuppressive T cells; therefore, not all patients with CRC may benefit from these treatments (20). Therefore, it is crucial to establish a characteristic classification for effectively targeting specific CRC subtypes and screening patients who may benefit the most from ICI treatment. Widely used biomarkers such as PD-L1 levels, TMB (tumor mutational burden), TIDE, and high microsatellite instability are not always reliable (21), highlighting the need to identify prognostic biomarkers for CRC immunotherapy.

In this study, 649 differential IRGs of CRC were selected from patient information to construct the IRGPI. The most relevant biological processes and signaling pathways were “humoral immune response” and “cytokine–cytokine receptor interaction,” respectively, which is consistent with the pathological process of the CRC immune response reported in the literature (22). IRGs were fitted into five modules, and 11 IRGs were screened out, confirmed the independent and effective prognostic factors, and performed OS analysis to verify this result. We also confirmed that IRGPI is closely associated with clinicopathological factors. Specifically, patients with CRC having a low IRGPI risk score showed a better prognosis, whereas those with a high IRGPI risk score had a worse prognosis.

After that, we found that the low-risk group had a higher mutation rate, whereas the largest difference in mutations between groups was in *TP53* mutations, which were more common in IRGPI-high samples than IRGPI-low samples (60% vs. 46%). As we all know, the TP53 mutation was linked to more aggressive disease and poorer patient outcomes in CRC through the p53/HRK/XEDAR signaling pathway (23, 24). Therefore, IRGPI-high patients with high *TP53* mutations have a worse outcome than IRGPI-low patients with low *TP53* mutations, in agreement with our survival results. Meanwhile, the more MMR in the low-risk group means more likely benefits from ICIs. The ICs related with the differential immune cells and immune function. Classically, CTLA-4 interacting with the B7 molecules, including PD-1 (programmed death-1), PD-L1 (programmed death ligand-1) (B7-H1), and PD-L2 (B7-DC), results in decreased T-lymphocyte activity and regulates the immune response (23, 24). Similarly, PD-1 interactions with PD-L1 and PD-L2 downmodulate T-cell immune responses (25). FOXP3 (Forkhead box protein 3), commonly used as a marker in Treg (regulatory T cells) cells, is an important transcription factor in the immunosuppressive function of CRC (26). LAG-3 (lymphocyte-activation gene 3) is associated with the immune resistance of CD4+ cells in patients with CRC (27). CD27 is a member of the TNF-receptor superfamily. This receptor plays a key role in regulating B-cell activation and immunoglobulin synthesis (28). According to the difference of the expression of these IC genes between the two IRGPI subgroups, we can conclude that ICs are closely related to the immune function of T cells and B cells.

In the next study, we found that macrophages, T helper cells, and type I IFN responses showed obvious higher immune function scores in the IRGPI-high group; meanwhile, there are significant differences in T cells between the IRGPI-high group and the IRGPI-low group. To further explore the key immune cell interactions that produced differences between the high- and low-risk groups, we performed differential immune cell-related and immune function-related K–M analyses. As a result, patients with more naïve B cells, M1 macrophages, and T-cell regulatory (Treg) infiltration had poor OS, whereas patients with more resting dendritic cells and activated mast immune cell infiltration had a longer OS. From the above results, we can draw a conclusion—the key immune cell interactions that produced different prognoses between the high- and low-IRGPI groups may be related to three immune cells: macrophages, T cells, and B cells. Therefore, in the next experiment, we selected these three immune cells for further study in a murine CRC model.

Immune cell infiltration is important for tumor progression; however, it is an underrated factor for evaluating the efficacy of ICI treatment (29). Increasing evidence has shown that the interaction between tumors and the microenvironment is critical for the progression of CRC and effectiveness of immunotherapy (30). Therefore, we assessed the relative proportions of 22 immune cells in the high- and low-risk subgroups of CRC samples, including immune cell infiltration and immune function scores and the roles of various immune cells to explain the low response to ICI in patients with CRC. In general, large numbers of activated memory CD4+ T cells, cytotoxic T cells, and CD8+ T cells contribute to the immune response and are associated with better prognosis (31), whereas resting immune cells indicate a state of immune failure (32). Interestingly, a recent study (33) described that mast cells play important roles in ICI therapy. We found that activated mast cells prolonged OS, suggesting a more active anti-tumor immune response in low-risk patients. Follicular helper T cells are a distinct subset of CD4+ helper T cells that activate B cells, generate specific antibody responses, and play important roles in the progression of autoimmune diseases (34). Follicular helper T cells suppress the development of regulatory B cells, indicating a poor prognosis for digestive system cancers (35). Therefore, high infiltration in the high-risk subgroup suggests an immunosuppressive tendency. High-risk patients with more naïve B cells, macrophages M1, and Tregs, which suppress the tumor immune response, have a shorter OS.

To understand the constitutive mechanism of the IRGPI coefficient, we explored the constitutive genes of IRGPI. RBCK1 not only reduces chemosensitivity (36) but also reduces lymphocyte activity *via* MALT1 (37). CD36 regulates cytokine production, antigen presentation, phagocytosis, and immune tolerance. Because inflammation triggers the initiation, proliferation, invasion, and metastasis of tumor cells, reducing CD36-mediated sterile inflammation may become a new mode of anti-tumor treatment (38). CD36 can also participate in tumor pathogenesis by regulating the PPAR pathway and inhibiting the mitochondrial biogenesis regulator gene *PPARGC1A* (39). Aberrant methylation of *TRIM58* has become a biomarker in multiple cancer prognostic models (40–42). IL1RL2 binding to IL-36 orchestrates an innate-adaptive immune linkage to control enteropathogenic bacterial infections (43) and promotes intestinal fibrosis in mice with chronic intestinal inflammation (44). MC1R promotes UV-induced DNA damage repair (45), and its frequent mutations are associated with an increased risk of CRC (46). In contrast, CCL24 is highly expressed in patients with CRC, is associated with a better prognosis (47), and specifically induces M1 macrophage chemotaxis (48). *LGALS2* is an oxidative stress-responsive gene that inhibits colon tumor growth (49). ADIPOQ is secreted by adipocytes in the tumor microenvironment, is widely present in the intestinal tract, and has been shown to activate cytotoxic autophagy induced by cancer cells; thus, elevated ADIPOQ levels are associated with decreased cancer growth (50). The results of these previous studies are consistent with those of the current study, showing that the correlation coefficients of immune-related oncogenes can be used when calculating IRGPI risk scores.

In summary, the IRGPI constructed based on 11 genes can accurately predict the survival rate of patients with CRC, reflect their immune microenvironment, and predict the sensitivity of immunotherapy. The mouse model of CRC constructed to observe IRGPI-related genes and immune cells in the CRC tumor microenvironment also showed differences in the expression of 11 IRGPI genes, supporting the validity of the IRGPI. Immune cells with immunosuppressive functions, including Tregs, M1 macrophages, and M2 macrophages, showed higher proportions in CRC mice than normal mice. Normal mice showed a higher proportion of active anti-tumor immune effector cells, such as B cells and memory B cells, and tended to have increased levels of helper T lymphocytes and cytotoxic T lymphocytes. These results confirm the expression changes of IRGPI genes in CRC tumor tissue and showed that the IRGPI accurately reflects the level of immune cell infiltration in CRC tumors.

Several biomarkers, such as TIDE and TIS, have been reported to predict patient responses to immunotherapy (51, 52). TIDE scores can predict prognosis more accurately compared to other biomarkers, such as PD-L1 levels and mutation burden, in patients with melanoma treated with first-line ICIs (15). Higher TIDE scores are associated with poorer outcomes (53). We observed higher TIDE scores in the IRGPI high-risk subgroup than in the IRGPI low-risk subgroup, suggesting that patients with low IRGPI benefited more from ICI treatment than those with high IRGPI. However, TIDE and TIS only focus on the function and state of T cells, which cannot fully reflect the complexity of immunocytes involved in immunotherapy in the TIME (54). Therefore, the IRGPI not only predicted differences in immune infiltration and immune function in the TIME of patients with CRC, but also showed a more accurate predictive ability than TIDE and TIS and better prediction of OS during long-term follow-up.

In conclusion, IRGPI is a promising immune-related prognostic marker that can intuitively predict the prognosis and immunotherapy effects in CRC (55, 56). In the era of precision medicine, biomarkers based on IRGs are expected to become effective tools for the clinical treatment of CRC. However, animal experiments, such as in mice, are needed to further evaluate the use of ICIs for treating CRC, observe and analyze the relationship between survival status and IRGPI-related genes and immune cell infiltration, and verify the effectiveness of IRGPI. Moreover, larger numbers of patients with CRC should be evaluated in prospective studies to validate and improve our approach.

## Data availability statement

The datasets presented in this study can be found in online repositories. The names of the repository/repositories and accession number(s) can be found below: https://figshare.com/, https://figshare.com/articles/dataset/data_for_IRGPI/19534810.

## Ethics statement

All experimental procedures were performed in accordance with the National Institutes of Health Guidelines for Laboratory Animals and approved by the Animal Ethics Committee of Nanjing University of Chinese Medicine (202010A026).

## Author contributions

DT and JG conceived the concept and critically reviewed the manuscript; ZL and PT collected and assembled the data and finished the manuscript; RS and YL performed the experiments; FL, LL, YB, MJ, FZ and GY provided experimental assistance. All authors contributed to the article and approved the submitted version.
