# Supplementary material for: Immune-related gene-based prognostic index for predicting survival and immunotherapy outcomes in colorectal carcinoma
Source: Front Immunol. 2022 Dec 13;13:944286. doi: 10.3389/fimmu.2022.944286 (PMC9795839; doi:10.3389/fimmu.2022.944286)
Supplement: Supplementary file 1 [file DataSheet_1.docx]

**Supplementary Data**

**Establishment and Identification of** **immune-related genes prognostic index for predicting survival and immunotherapy in colorectal carcinoma**

Zhongqing Liang^1#^, Ruolan Sun ^1#^, Pengcheng Tu^2,4#^, Yan Liang ^1^, Li Liang^1^, Fuyan Liu^1^, Yong Bian^1,3^, Gang Yin^1^, Fan Zhao^1^, Mingchen Jiang^1^, Junfei Gu^1*^, Decai Tang^1*^

**Affiliations**

1. School of Chinese Medicine, School of Integrated Chinese and Western Medicine, Nanjing University of Chinese Medicine, Nanjing, Jiangsu 210023, China

2. Affiliated Hospital of Nanjing University of Chinese Medicine, Nanjing, 210029, China

3. Laboratory Animal Center, Nanjing University of Chinese Medicine, Nanjing, 210023, China.

4. Laboratory of New Techniques of Restoration & Reconstruction of Orthopedics and Traumatology, Nanjing University of Chinese Medicine, Nanjing, Jiangsu 210023

⁎**Corresponding author:**

Decai Tang, Nanjing University of Chinese Medicine, Nanjing, No. 138 Xianlin Avenue, 210023, China. E-mail address: talknow@njucm.edu.cn

Junfei Gu, Nanjing University of Chinese Medicine, Nanjing, No. 138 Xianlin Avenue, 210023, China. E-mail address: gujunfei@njucm.edu.cn

^#^These authors contributed equally to the study.

**Supplementary Figures**

**Figure S1**

**
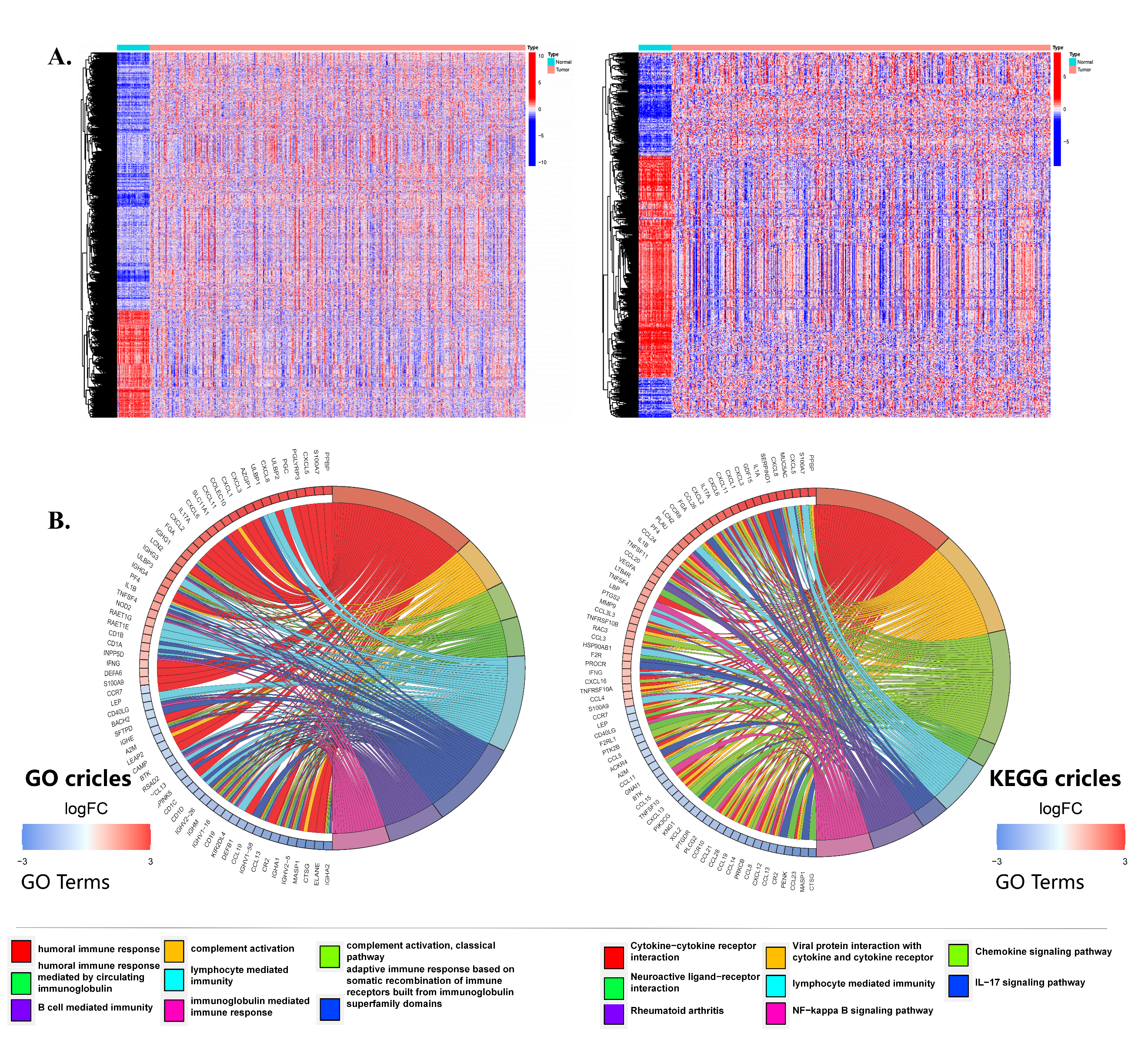
**

**Fig. S1. Differentially expressed immune‐related genes in CRC.**

**(A)** Heatmap displaying all differentially expressed genes (DEGs) between 473 CRC samples (Coral) and 41 para-cancer samples (Turquoise); Heatmap displaying immune-related DEGs between 473 HNSCC samples (Coral) and 41 para-cancer samples (Turquoise).

**(B)** Visualization by Circles for Gene Ontology (GO) and Kyoto Encyclopedia of Genes and Genomes (KEGG) enrichment analysis results of the immune-related DEGs (p < 0.05).

**Figure S2**

**
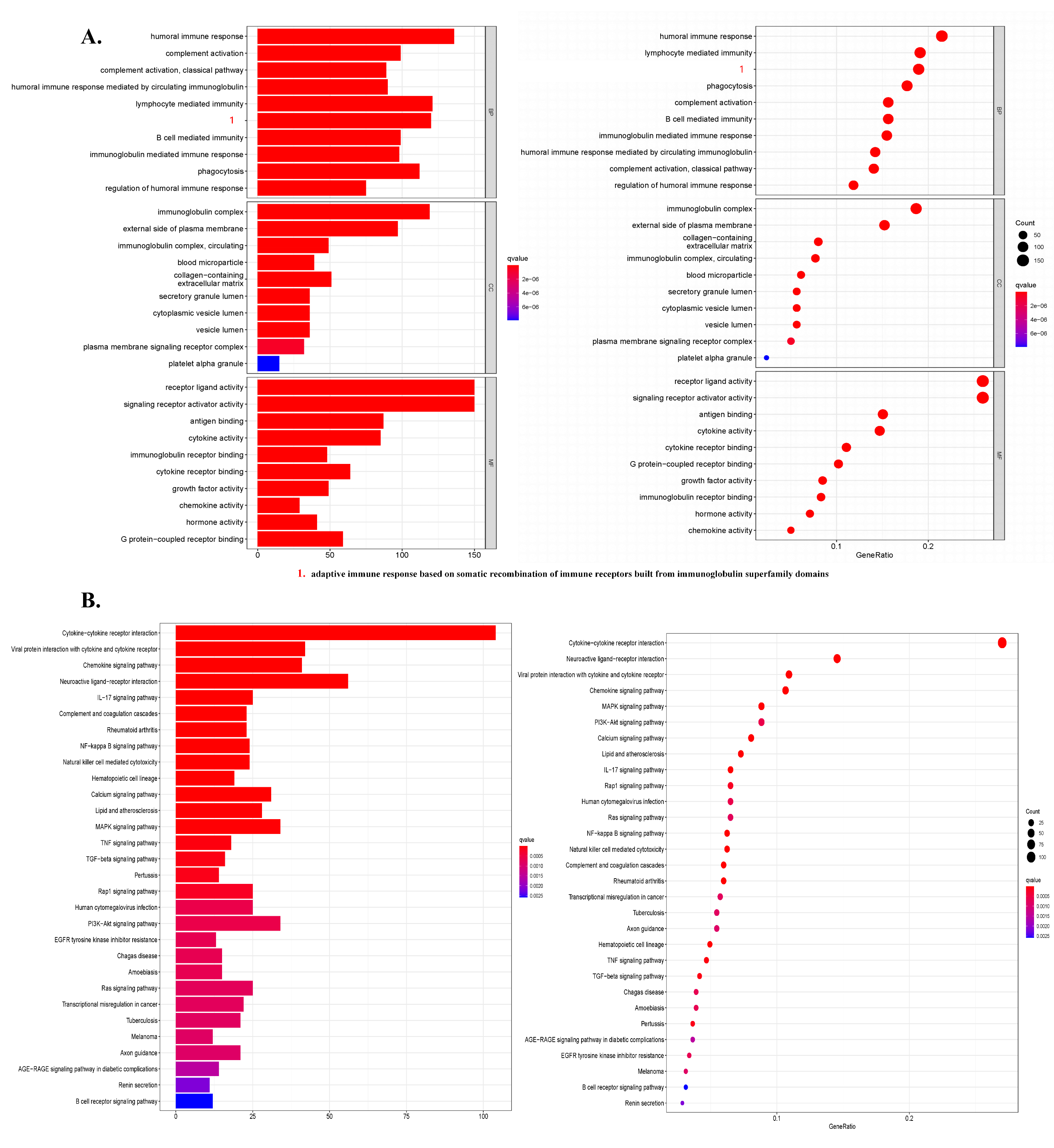
**

**Fig. S2. GO terms and KEGG pathways enrichment analysis of Top 30.**

**(A)** Bar and Bubble plots of GO terms enrichment analysis of the immune-related DEGs in BP, CC and MF (Top 10, P adjust < 0.01), respectively. **(B)** Bar and Bubble plots of KEGG pathways analysis of the immune-related DEGs (Top 30, P adjust < 0.01).

**Figure S3**


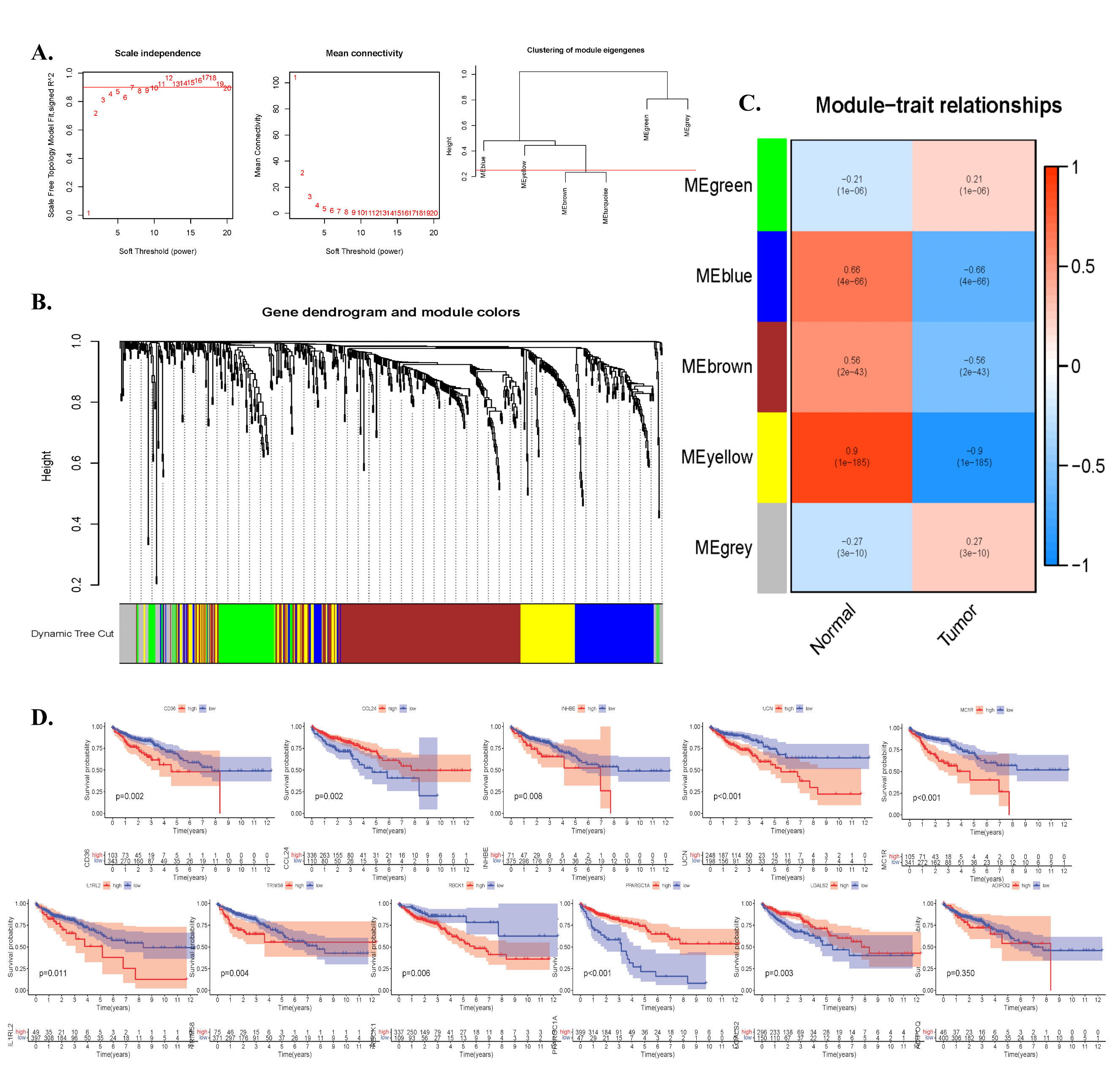


**Fig. S3. Results of** **weighted gene co-expression network analysis (WGCNA).**

**(A)** Determination of the soft-thresholding power in the WGCNA analysis. The scale-free fit index of various soft-thresholding powers (Soft-Power = 4); Mean connectivity of various soft-thresholding powers; Clustering of similar modules (MEDissThres = 0.25).

**(B)** A dendrogram of the differentially expressed genes clustered based on different metrics.

**(C)** Heatmap of associations between module eigengenes of normal and tumor tissues.

**(D)** Kaplan-Meier survival analysis of the 11 immune-related hub genes that used to compute IRGPI risk score.

**Figure S4**


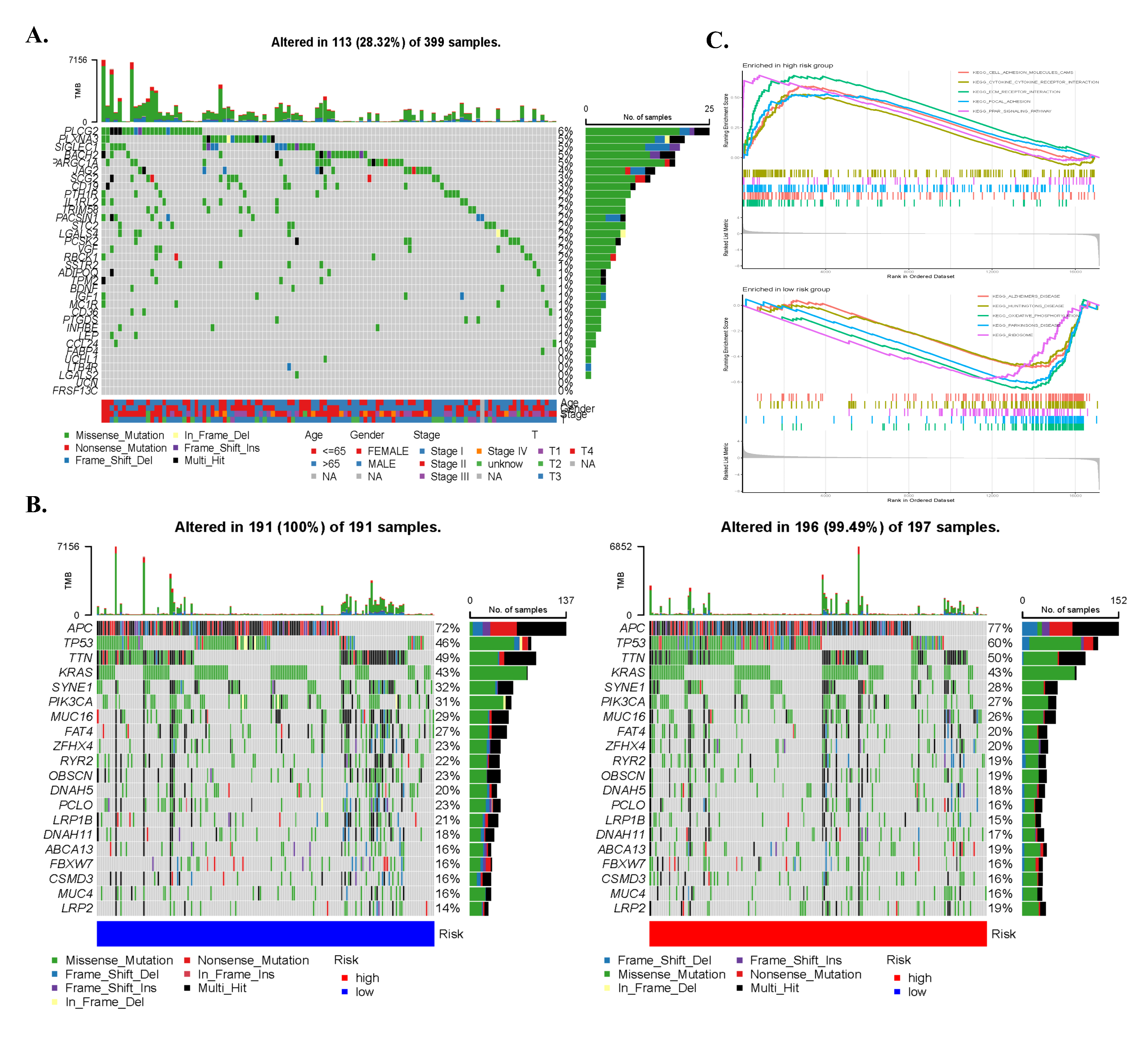


**Fig. S4. Oncoplot for somatic mutation and GSEA enrichment**

**(A)** Oncoplots for somatic mutation of 34 immune-related hub genes with that used for Univariate Cox regression in 399 samples.

**(B)** Oncoplots for top ten mutated genes between the IRGPI low-risk(blue) and high-risk subgroups(red).

**(C)** GSEA pathway enrichment between the low- and high- risk IRGPI subgroups.

**Figure S5**


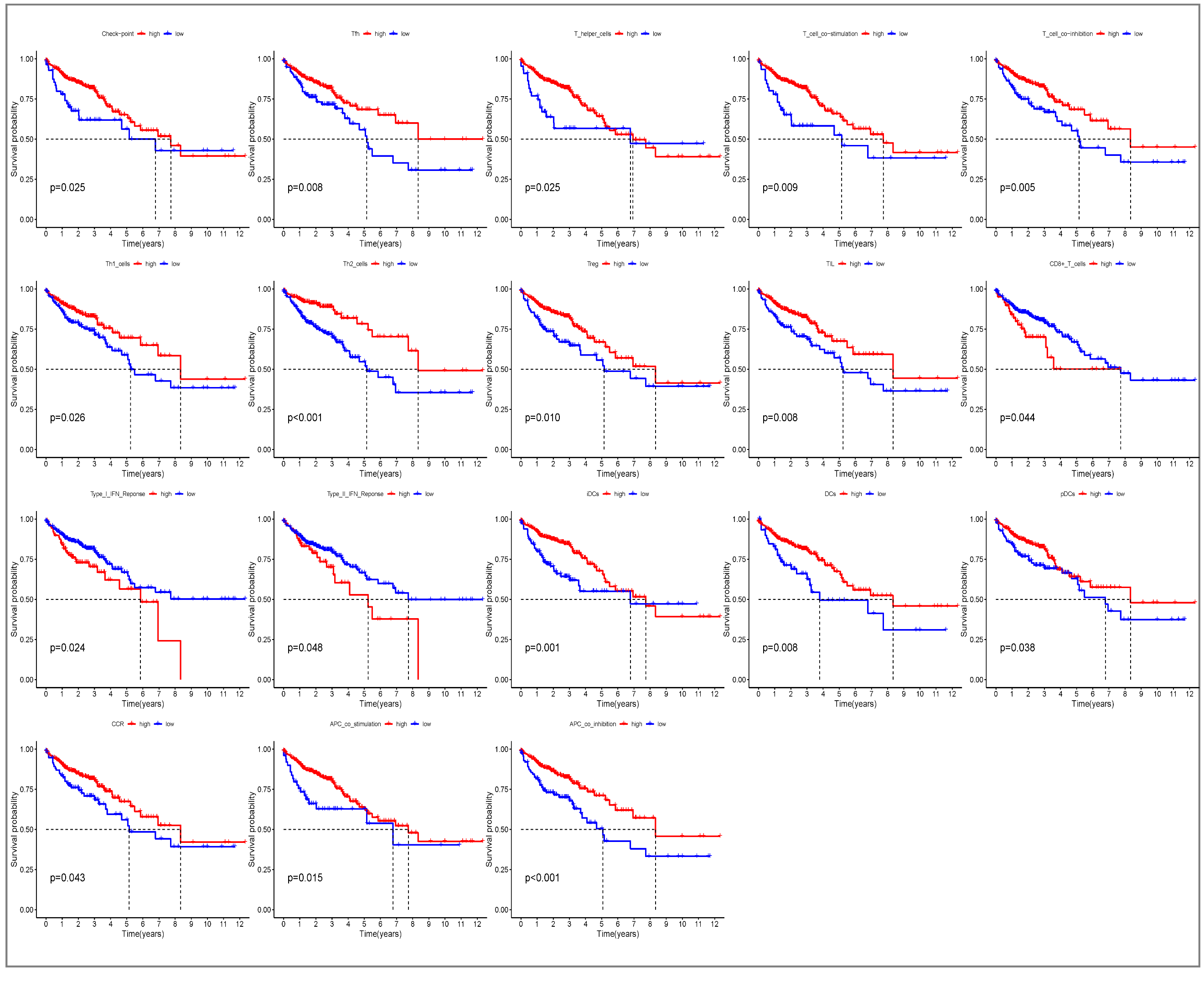


**Fig. S5. Kaplan-Meier survival analysis of the immune function scores of IRGPI subgroups.**

**Figure S6**


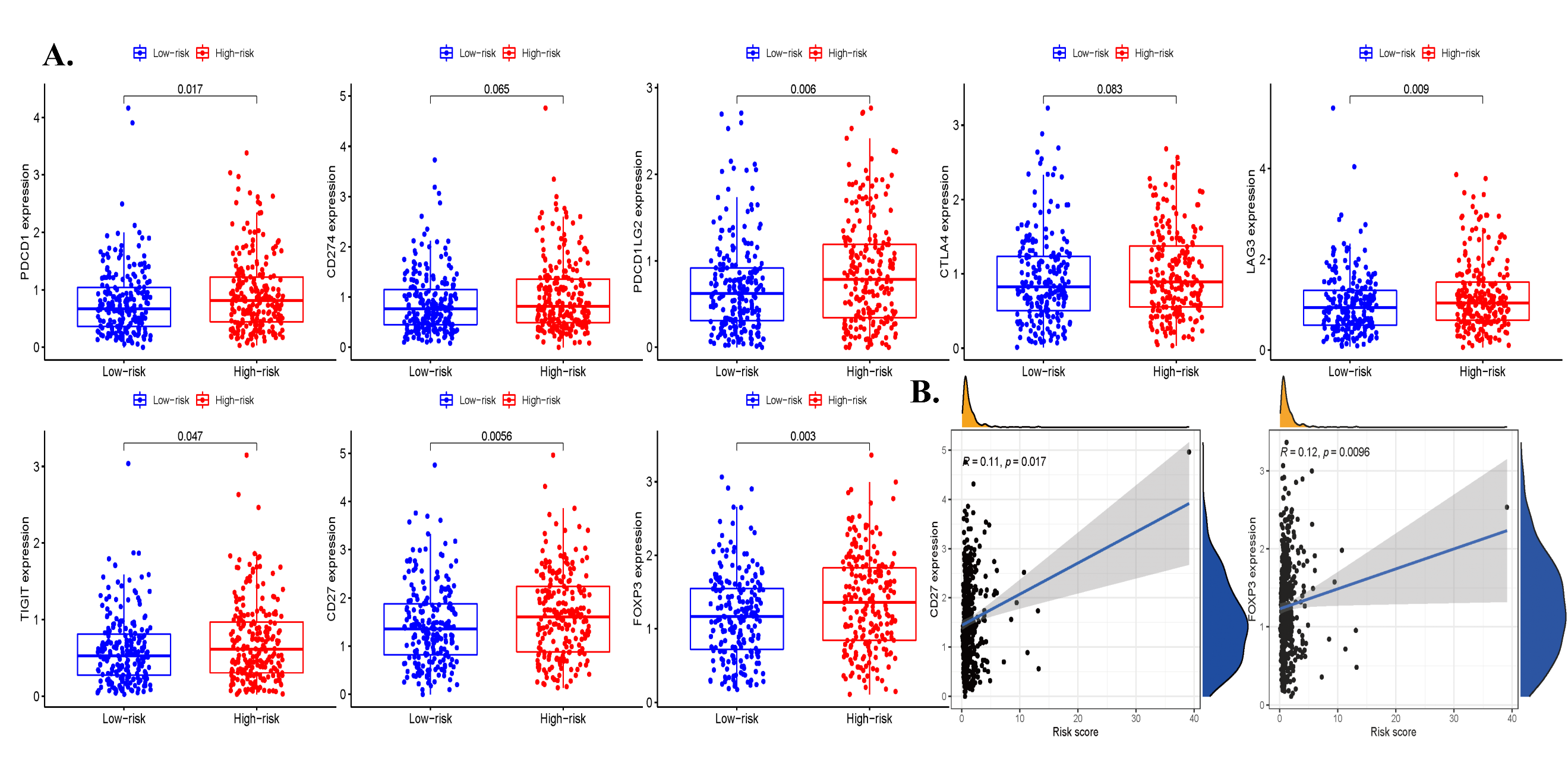


**Fig. S6. IRGPI signifcantly correlates with multiple clinicopathological factors in CRC patients and the analysis of immunotherapy responses.**

**(A)** Scatter plots of Immune Checkpoint genes PD-1, PD-L1, PD-L2, CTLA4, LAG3, TIGIT, CD27, FOXP3 expressions between IRGPI subgroups.

**(B)** Scatter plots coordinated by IRGPI risk score with Immune Checkpoint CD27, FOXP3 respectively.

**Figure S7**


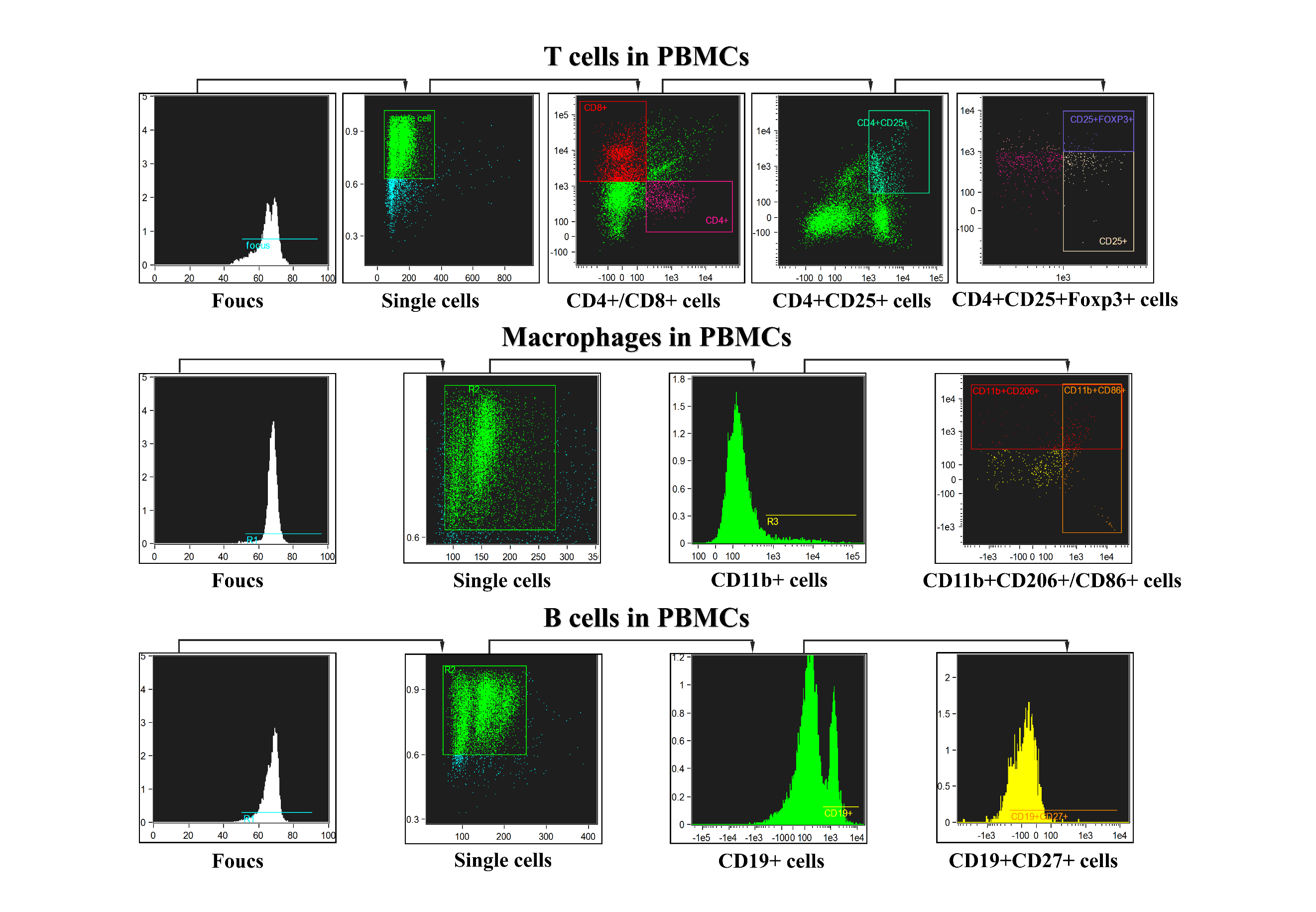


**Fig. S7. Analysis strategies of Flow cytometry determined IRGPI related-immunocyte subsets.**

**Supplementary Tables**

**Table S1. 452 CRC TCGA-CODA cases**

| **Data Set**  **Cases** | **TCGA-COAD** | **Data Set**  **Cases** | **TCGA-COAD** |
| --- | --- | --- | --- |
|  | **(n=452)** |  | **(n=452)** |
| **Age** [year] | 31 - 90 | **Vital Status** |  |
| **Gender** [n (%)] |  | Alive | 354(78.0%) |
| Male  Female | 238(52.4%)  214(47.1%) | Dead | 98(21.6%) |
|  |  | Unknown | 2(0.44%) |
| **Race** [n (%)] |  | **Disease Type** [n (%)] |  |
| White | 211(46.5%) | Adenomas and adenocarcinomas | 387(85%) |
| Not reported | 171(37.7%) | Cystic, mucinous and  serous eoplasms | 64(14.1%) |
| Black or african american | 58(12.8%) |  |  |
| Asian | 11(2.4%) | Complex epithelial neoplasms | 2(0.45%) |
| American indian or alaska native | 1(0.2%) | Epithelial neoplasms, nos | 1(0.2%) |

**Table S2. CRC TCGA-CODA and GSE161158 cases of clinical files**

| **Data Set**  **Cases** | **TCGA-COAD**  **(n=452)** | **GSE161158**  **(n=250)** | **Data Set**  **Cases** | **TCGA-COAD**  **(n=452)** | **GSE161158**  **(n=250)** |
| --- | --- | --- | --- | --- | --- |
| **Age** [year,$\overline{x}\pm s$] | 64±13.23 | 67±13.00 | **T** [n (%)] |  |  |
| **Gender** [n (%)] |  |  | T1 | 10(2.2%) | _ |
| Female  Male | 214(47.3%)  238(52.7%) | _  _ | T2 | 77(17%) | _ |
|  |  |  | T3 | 308(68.1%) | _ |
| **Stage** [n (%)] |  |  | T4 | 56(12.3%) | _ |
| I  II  III  IV  Unknown | 76(16.8%)  178(39.4%)  125(27.7%)  62(13.7%)  10(2.2%) | 33(13.2%)  76(30.4%)  82(32.8%)  59(23.6%)  0(0%) | Tis | 1(0.2%) | _ |
|  |  |  | **M** [n (%)] |  |  |
|  |  |  | M0 | 334(73.9%) | _ |
|  |  |  | M1 | 62(13.7%) | _ |
|  |  |  | MX | 48(10.6%) | _ |
| **N** [n (%)] |  |  | Unknown | 7(1.5%) | _ |
| N0 | 269(59.5%) | _ | **Vital Status** |  |  |
| N1 | 103(22.8%) | _ | Alive | 356(78.8%) | 145(0.58) |
| N2 | 80(17.7%) | _ | Dead | 96(21.2%) | 59(23.6%) |
| Unknown | 0(0%) | _ | Unknown | 0(0%) | 46(18.4%) |

**Table S3.** **Univariate Cox regression of 34 immune-related hub genes.**

| **FABP4** | **ADIPOQ** | **LEP** | **TPM2** | **BDNF** | **IGF1** | **SCG2** | **UCHL1** | **CD36** |
| --- | --- | --- | --- | --- | --- | --- | --- | --- |
| **PTGDS** | **BACH2** | **PCSK2** | **CCL24** | **CD19** | **PLCG2** | **INHBE** | **UCN** | **VGF** |
| **IL1RL2** | **PTH1R** | **SSTR2** | **TNFRSF13C** | **TRIM58** | **PACSIN1** | **SIGLEC1** | **RBCK1** | **LTB4R** |
| **PLXNA3** | **JAG2** | **STC2** | **MC1R** | **PPARGC1A** | **LGALS2** | **LGALS4** |  |  |

**Table S4. IRCPI - related murine genes Primer sequences used in qRT-PCR**

| Gene | Forward Primer (5' - 3') | Reverse Primer (3' - 5') | Amplicon Size |
| --- | --- | --- | --- |
| *Adipoq* | TGTTCCTCTTAATCCTGCCCA | CCAACCTGCACAAGTTCCCTT | 104 |
| *Cd36* | ATGGGCTGTGATCGGAACTG | GTCTTCCCAATAAGCATGTCTCC | 110 |
| *Ccl24* | ATTCTGTGACCATCCCCTCAT | TGTATGTGCCTCTGAACCCAC | 187 |
| *Inhbe* | AAAAGCCCAGCTCTGGCTAAT | CTGGTTAGGTGCAGTCCCTC | 165 |
| *Ucn* | TCTTGCTGTTAGCGGAGCG | TCGAATATGATGCGGTTCTGC | 181 |
| *Il1rl2* | GCAGCAGATACGTGTGAGGAC | GTACCATGTCAGATTTACTGCCC | 120 |
| *Trim58* | TTGACACGATGTGAGGCTATCA | GTCCGCAGTTAAGAGCAAGC | 165 |
| *Rbck1* | CTGCTATCAAGTATGCCACCTG | TGTGCATGTACGCATCCTCC | 126 |
| *Mc1r* | AGAGCCTTGGTGCCTGTATG | TGACACTTACCATCAGGTCAGAC | 176 |
| *Ppargc1a* | TATGGAGTGACATAGAGTGTGCT | CCACTTCAATCCACCCAGAAAG | 134 |
| *Lgals2* | AACATGAAACCAGGGATGTCC | CGAGGGTTAAAATGCAGGTTGAG | 116 |
